# Physical exercise and academic procrastination in Chinese college student-athletes: the chain mediating role of peer support and academic self-efficacy

**DOI:** 10.3389/fpsyg.2026.1813953

**Published:** 2026-05-25

**Authors:** Maqiang Chen, Kunlan Zhan, Yuan Song

**Affiliations:** 1 Department of Physical Education, Chongqing University of Technology, Chongqing, China; 2 School of Management, Chongqing University of Technology, Chongqing, China

**Keywords:** academic procrastination, academic self-efficacy, peer support, physical exercise, student-athletes

## Abstract

**Objective:**

Grounded in Bandura's Social Cognitive Theory, particularly the model of triadic reciprocity, this study investigates the mechanisms through which physical exercise influences academic procrastination through the chain mediation of peer support and academic self-efficacy among Chinese college student-athletes.

**Methods:**

332 Chinese college student-athletes participated in the study. Physical exercise, peer support, self-efficacy, and academic procrastination were assessed using the Physical Activity Rating Scale-3, Peer Support Scale, Academic Self-Efficacy Scale, and Procrastination Assessment Scale-Students, respectively. Pearson correlation analysis, linear regression analysis, and mediation analysis were used to analyze the data.

**Results:**

The results indicated that, among all the mediation paths examined, only the sequential mediation path physical exercise → peer support → academic self-efficacy → academic procrastination reached statistical significance.

**Conclusion:**

These findings demonstrates that physical exercise may reduce academic procrastination behavior among student-athletes primarily through a sequential pathway in which enhanced peer support strengthens academic self-efficacy. In addition, the comparison between SPSS PROCESS and AMOS indicates that different analytical approaches may yield different mediation results, highlighting the importance of carefully selecting and reporting statistical methods in future research on chained mediation models.

## 1 Introduction

Student-athletes represent a distinct group within the collegiate population. The National Collegiate Athletic Association (NCAA) defines them as full-time students enrolled in a 4 year institution who have joined a varsity team before the semester begins and participate in its training and competitions. In contrast, China lacks a uniform definition of student-athletes. In the Chinese context, the term generally refers to students who receive formal higher education while also undergoing systematic sports training and participating in competitive events (Huang et al., 2022). Despite their athletic involvement, student-athletes primarily identify as students, with athletic participation representing an extension of their talents; therefore, they are required to balance athletic training with academic responsibilities. Given the dual responsibilities of academic study and athletic training, student-athletes often face considerable academic pressures and time-management challenges. Among these challenges, academic procrastination has emerged as a common behavioral issue that may affect their academic performance and psychological wellbeing.

Academic procrastination refers to the voluntary delay of an intended course of action despite awareness of likely negative consequences (Steel, 2007). Existing studies have shown that academic procrastination exerts multiple adverse effects on students, including declined academic performance, exacerbated negative emotions such as depression and anxiety, impaired sleep quality, and reduced sense of wellbeing and life satisfaction (Sirois et al., 2019; Sirois and Kitner, 2015). Due to its severe repercussions, academic procrastination has evolved from an individual behavioral issue into a common educational challenge requiring policy intervention. In 2023, a document titled “Opinions on Deepening the Classified Development of Academic and Professional Degree Graduate Education” was issued, which stressed improving the academic early-warning, the reassignment and withdrawal mechanisms for postgraduate students. It called for regular academic alerts based on students' actual training status and the timely streamlining or withdrawal of graduate students deemed unsuited to continue their studies in their respective disciplines, so as to ensure the quality of postgraduate training. In 2025, many Chinese universities have released academic early-warning lists and implemented measures such as warnings, academic demotion, and advised withdrawal warnings to remind and urge students to complete their academic tasks in a timely manner. Thus, it is evident that academic procrastination not only profoundly undermines students' physical and mental health but also erects structural barriers to their practical development, posing a direct threat to their academic careers.

Current research on academic procrastination among university student-athletes remains limited. A representative study by Gaston-Gayles (2004) identified the inherent conflict between academic and athletic identities as a fundamental challenge. This institutionalized role contradiction makes it difficult for student-athletes to balance their limited psychological resources, thereby constituting a profound risk factor for academic procrastination. Research in the Chinese context further demonstrates that the role conflict stemming from the “athlete-student” duality significantly consumes individuals' psychological resources, consequently exacerbating academic procrastination (Ren et al., 2021). Notably, psychological factors play a crucial role in this process: existing research confirms that self-regulatory capacity serves as an important predictor of academic performance among student-athletes, and deficiencies in this capacity directly elevate levels of academic procrastination (Krug et al., 2021). Therefore, conducting an in-depth analysis of the psychological mechanisms underlying academic procrastination among college student-athletes and developing targeted intervention strategies are of paramount importance for ensuring their academic success and sustainable career development.

Self-regulation theory, proposed by American psychologist Albert Bandura in the 1980s, describes a process in which individuals set personal behavioral standards and reinforce, maintain, or adjust their own behaviors through self-administered rewards or punishments. The core characteristic of this theory is that individuals monitor and adjust cognitive elements such as attitudes and subjective norms, along with behavioral drivers like desires and intentions, thereby narrowing the gap to their goals to achieve expected outcomes. Its central focus lies in executive functions, impulse control, and resource management (Baumeister et al., 1998). The theory holds that, specifically, in the pathway of “dysfunctional beliefs → desire-driven → sensitivity to delay → voluntarily delay,” individuals may experience delays in task execution due to insufficient self-management capabilities (Rozental et al., 2015). Physical exercise is a structured physical activity designed to enhance physical and mental health (Carrapatoso et al., 2022). Studies have shown that physical exercise can significantly enhance aerobic fitness, improve students' attentional inhibition, cognitive flexibility, and related brain functions (Hillman et al., 2014). Existing research has confirmed a significant negative correlation between physical exercise and academic procrastination (Hu et al., 2025; Zhuan et al., 2024). Currently, studies on the impact of physical exercise on academic procrastination have mostly focused on middle school students or the general college student population, while specialized research targeting student-athletes remains insufficiently evidenced. The dual demands of academics and training deplete student-athletes' self-regulatory resources, leading to procrastination through diminished focus and poor goal management. Physical exercise counteracts these deficits by enhancing neural mechanisms underlying executive function-replenishing self-regulation capacity and strengthening goal adherence. Gollwitzer and Sheeran (2006) confirm that exercise improves self-regulatory dimensions including attitude monitoring, normative judgment, desire-driven motivation, and intention implementation, demonstrating a significant negative association with academic procrastination. It is therefore hypothesized that physical exercise may mitigate academic procrastination among student-athletes by enhancing their self-regulatory capacities. Based on this, this study proposes Hypothesis 1: Physical exercise has a significant negative predictive effect on the academic procrastination among student-athletes.

Peer relationships refer to interpersonal relationships formed and developed through interactions among individuals of similar age or comparable psychological maturity, encompassing two primary dimensions: peer acceptance, and friendship quality (Rubin et al., 2007). The concept of peer support can be traced back to the late 1970s. Through ongoing development and refinement, scholars subsequently established its core principles and values. Mead et al. (2001) defined it as a system of mutual aid based on respect, shared responsibility, and reciprocal understanding. Rather than relying on psychiatric models or diagnostic criteria, this framework fosters empathetic understanding of personal circumstances through shared experiences of emotional and psychological distress. Evidence from a number of recent studies has shown a clear link between peer support and academic procrastination. Patria and Laili (2021) used a 15 day writing group with peer support and found that it significantly reduced thesis procrastination in graduate students. Looking more closely at how this works, Jin et al. (2019) discovered that when college students feel closely connected to their peers, it helps them build greater perseverance, which in turn reduces procrastination. Studying the situation during the pandemic, Melgaard et al. (2022) observed that students who tended to procrastinate struggled more with motivation and were less satisfied with their learning in online settings where peer support was missing.

According to Self-Determination Theory (SDT) (Deci and Ryan, 1985), peer support may reduce academic procrastination by satisfying students' three basic psychological needs: autonomy, competence, and relatedness (Ryan and Deci, 2000). By fulfilling these needs, peer support may enhance student-athletes' intrinsic motivation toward academic tasks and promote self-determined behavior, thereby reducing academic procrastination. It is therefore hypothesized that peer support exhibits a significant negative correlation with academic procrastination among student-athletes. Based on this, this study proposes Hypothesis 2: Peer support plays a mediating role between physical exercise and academic procrastination among student-athletes.

**Figure 1:**
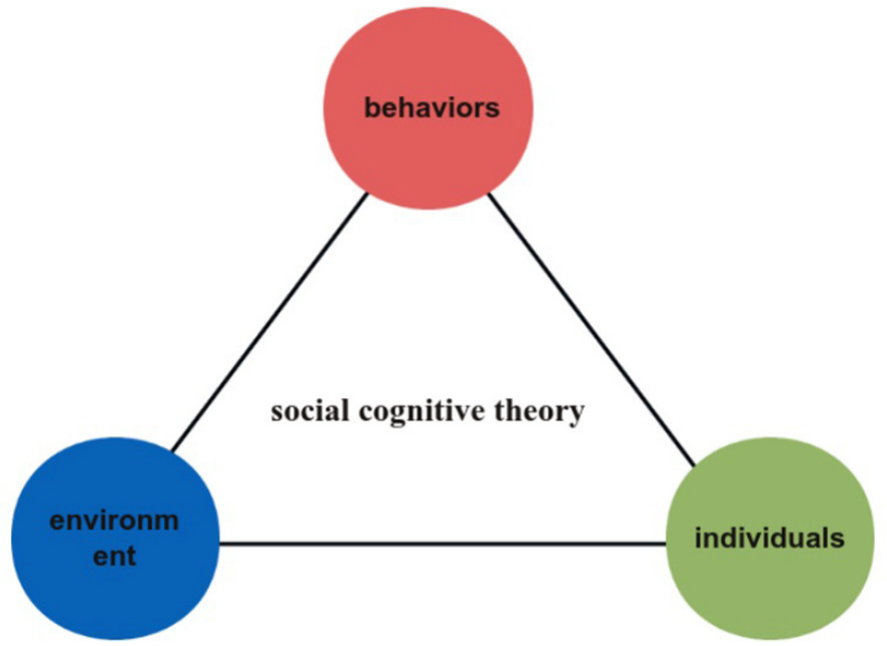
Triadic reciprocal model of social cognitive theory.

Academic self-efficacy, a concept officially proposed by Bandura in 1986, refers to an individual's subjective judgment regarding their ability to accomplish a specific learning goal (Bandura, 1986). In the same year, social cognitive theory was proposed in his work Social Foundations of Thought and Action, which focuses on the triadic reciprocal interaction among individuals, behaviors, and the environment (Bandura, 2006), as illustrated in Figure 1. The theory emphasizes the predominant role of self-efficacy in behavioral choices and motivational intensity. Its operational mechanism follows this logic: after individuals form ability judgments through past experiences and vicarious learning, individuals with high self-efficacy develop positive outcome expectations and persistence in action, whereas those with low self-efficacy are prone to exhibit avoidance and procrastination (Bandura, 2006). For student-athletes, strong academic self-efficacy fosters confidence in balancing academic and athletic commitments, leading to proactive planning and reduced procrastination. Conversely, low self-efficacy triggers anxiety and avoidance behaviors that promote task delay. Meanwhile, environmental factors such as training intensity and team support continually shape their self-efficacy levels, while resulting academic setbacks further reinforce negative self-perceptions, thereby completing a cyclical pattern of procrastination. Substantial empirical evidence has established a well-documented relationship between academic self-efficacy and academic procrastination. Wolters (2003) pioneered the examination of academic procrastination within a self-regulated learning framework, revealing a significant association with students' academic self-efficacy. As mentioned earlier, Steel identified academic self-efficacy as one of the strongest and most stable predictors of academic procrastination. Klassen et al. (2008) further refined this relationship, demonstrating a significant negative correlation between academic self-efficacy and academic procrastination among college students. However, this association was weaker than that observed for self-regulatory efficacy. Wäschle et al., 2014 found that high self-efficacy and low procrastination form a beneficial cycle of “high goal achievement → stronger self-efficacy,” whereas low self-efficacy and high procrastination create a vicious spiral of “low goal achievement → diminished self-efficacy.” Based on this, this study proposes Hypothesis 3: Academic self-efficacy plays a mediating role between physical exercise and academic procrastination among student-athletes.

For college student-athletes, physical exercise serves not only as essential training to maintain competitive fitness but also as a central context for building peer interaction. The team-based training model frequently fosters collaboration and communication among teammates, thereby acting as a crucial catalyst for peer support. Academic procrastination, meanwhile, represents a typical behavioral issue for this group under the dual pressures of demanding training schedules and limited study time. Post-training fatigue can impair concentration, while rigorous training commitments often occupy time otherwise available for academics, which can easily trigger task-avoidance behaviors and exacerbate procrastination. In this process, peer support is a key environmental variable that can extend the cooperative relationships formed during training into the academic domain. Concurrently, academic self-efficacy, as a core individual cognitive factor, directly influences one's willingness to engage with academic tasks. Together, they form a pathway linking physical exercise and academic procrastination.

The triadic reciprocal model of social cognitive theory provides the core explanatory framework for the serial mediation pathway proposed in this study. The model posits that individual cognitive factors, behavior, and the environment are not unidirectional influences but rather dynamic interactions characterized by mutual shaping. First, physical exercise, as a “behavior” actively initiated by student-athletes, possesses inherent team-based characteristics that foster frequent collaboration and communication during training. This behavioral interaction establishes the necessary conditions for peer engagement, thereby enhancing “peer support” as an environmental factor and realizing the “physical exercise → peer support” pathway. Second, peer support as an environmental factor interacts with academic self-efficacy as a cognitive factor. Environmental selection and construction affect the bidirectional interactions among personal, behavioral, and contextual elements (Bandura, 1999). Specifically, academic assistance from peers serves as alternative experience, while their encouragement and recognition act as social reinforcement. Together, these mechanisms operate at the cognitive level to enhance academic self-efficacy, thereby completing the “peer support → academic self-efficacy” linkage. Finally, the cognitive factor of academic self-efficacy interacts with the academic procrastination. Among the self-referent thoughts that affect human motivation, affect, and action, none is more central or pervasive than people's judgments of their own efficacy (Bandura, 1999). Alternative experience serves as one of the most powerful means to create and strengthen beliefs in personal efficacy. Observing others with similar attributes achieve success through sustained effort strengthens individuals' belief in their own potential for achievement. Consequently, robust academic self-efficacy motivates student-athletes to proactively formulate post-training study plans, thereby reducing procrastination. This process establishes a complete chain mediation model in which physical exercise fosters peer support, which in turn enhances academic self-efficacy, and ultimately leads to a reduction in academic procrastination.

Previous research supports the links in this proposed chain. Specifically, physical activity effectively enhances students' perceived peer support (Xing and Ge, 2025). This support, in turn, serves as a key factor in fostering their academic self-efficacy (Altermatt, 2019). However, academic procrastination itself can also reversely erode students' academic self-efficacy by accumulating academic pressure and failure experiences (Wentzel et al., 2021) and diminish their willingness to seek support. This process entraps them in a vicious cycle of resource depletion (Hobfoll et al., n.d.). Furthermore, subsequent research has repeatedly confirmed a robust negative association between academic self-efficacy and academic procrastination (Klassen et al., 2008; Steel, 2007). Therefore, a compelling pathway is evident: physical exercise promotes peer support, thereby bolstering academic self-efficacy, which ultimately helps students mitigate academic procrastination. Based on this, the present study proposes Hypothesis 4: Peer support and academic self-efficacy play a chain-mediating role between physical exercise and academic procrastination among student-athletes.

**Figure 2:**
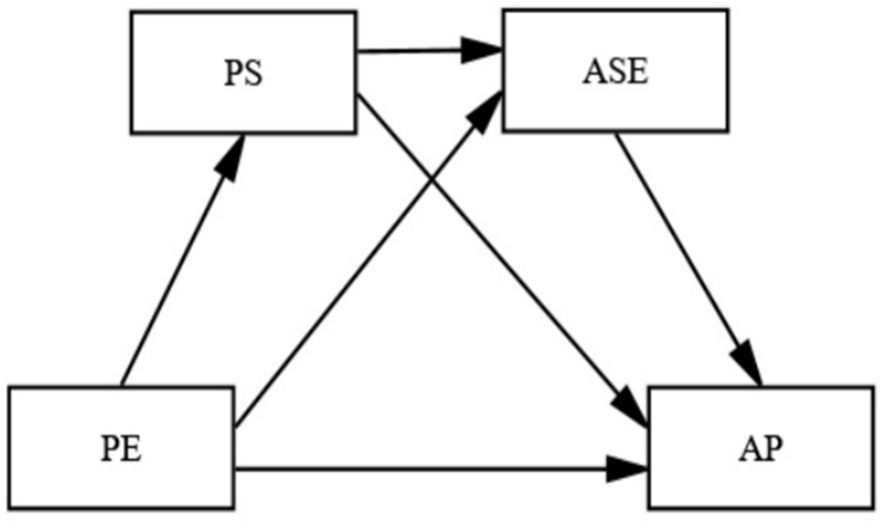
Proposed serial mediation model linking physical exercise, peer support, academic self-efficacy, and academic procrastination.

Grounded in Bandura's Social Cognitive Theory, particularly the model of triadic reciprocity, this study investigates the mechanisms through which physical exercise influences academic procrastination among Chinese college student-athletes. The triadic reciprocity framework posits that behavior, cognitive factors, and environmental influences interact as reciprocal determinants of one another. In this context, we conceptualize physical exercise as a core behavior, academic self-efficacy as a pivotal cognitive factor, and peer support as a critical environmental influence. We propose that physical exercise, as a health-promoting behavior, does not merely correlate with reduced academic procrastination but exerts its influence through a cascading social-cognitive mechanism. Specifically, the environmental support fostered within exercise settings is thought to enhance supportive peer networks, which in turn strengthens the cognitive belief in one's academic capabilities, thereby promoting proactive academic behaviors and reducing avoidance (see Figure 2).

Furthermore, testing this model within the unique population of Chinese student-athletes holds significant practical value. These individuals navigate the demanding dual roles of academic pursuit and athletic training, often facing acute time conflicts and performance pressure. The collectivistic nature of Chinese culture, which emphasizes group harmony and interdependence, may amplify the role of peer support as an environmental resource, making this population an ideal context for observing the triadic interaction between behavior, environment, and cognition. To isolate the relationships between our core constructs, we included gender, academic level, sport type (individual vs. team), and competitive level as control variables.

In summary, based on the foregoing, this study proposes the following hypotheses:

*H1:* Physical exercise will have a significant negative predictive effect on the academic procrastination among student-athletes.

*H2:* Peer support will play a mediating role between physical exercise and academic procrastination among student-athletes.

*H3:* Academic self-efficacy will play a mediating role between physical exercise and academic procrastination among student-athletes.

*H4:* Peer support and academic self-efficacy will play a chain-mediating role between physical exercise and academic procrastination among student-athletes.

## 2 Materials and methods

### 2.1 Participants and procedure

This study was conducted between November and December 2025. We employed a cluster random sampling method to recruit student-athletes from eight universities in China: Hainan Tropical Ocean University, Hunan Institute of Science and Technology, Tsinghua University, China University of Petroleum, Dalian University, Southwest University, Chongqing University of Science and Technology, and Chongqing University of Technology. A total of 416 questionnaires were distributed. In the present study, student-athletes were operationally defined as full-time university students who either engaged in systematic training with university sports teams to prepare for collegiate competitions at different levels or were physical education majors involved in competitive athletic preparation. Accordingly, the inclusion criteria were as follows: participants had to be full-time university students and either members of a university sports team or physical education majors involved in competitive athletic preparation.

The exclusion criteria included students who were neither sports majors nor members of a university sports team, as well as questionnaires with incomplete or invalid responses. To ensure sample homogeneity and data quality, a standardized screening procedure was implemented. Consistent with the study's primary objective, 84 respondents who were neither sports majors nor members of a university varsity team were excluded, thereby restricting the analytical sample to strictly defined student-athletes. Following this step, 332 valid questionnaires were retained, yielding a valid response rate of 79.8%. To ensure that the sample size was adequate for the statistical analyses conducted in this study, a priori power analysis was performed using GPower 3.1.9.2. Based on the most complex regression model in the study, which included 11 predictors, and assuming a medium effect size (*f*^2^ = 0.15), a significance level of α = 0.05, and a statistical power of 0.80, the minimum required sample size was estimated to be 123 participants. The final sample size of 332 therefore exceeded this requirement and was considered sufficient for the analyses conducted in this study. The final sample consisted of 259 male (78.0%) and 73 female (22.0%) full-time university student-athletes. Participants represented diverse sports and training backgrounds, supporting the representativeness of the sample for the target population. The study received ethical approval from the Ethics Committee of Chongqing University of Technology (Approval No. 20250096). Given that this study is a non-interventional, anonymous questionnaire survey, the committee waived the requirement for written informed consent accordingly. All participants were fully informed of the study's purpose, data confidentiality measures, and their right to withdraw at any time without consequence before completing the questionnaire. Data were collected online over approximately seven weeks using the Wenjuanxing platform. No compensation was provided for participation.

### 2.2 Measures

Standardized and widely used measurement scales with established reliability and validity were adopted to assess the key variables in this study: the independent variable (physical exercise), the dependent variable (academic procrastination), and the mediating variables (peer support and academic self-efficacy).

#### 2.2.1 Physical Activities Readiness Scale (PARS-3)

Physical exercise was assessed by the Physical Activity Rating Scale (PARS-3), developed by Hashimoto Kimio and revised by Liang (1994), consists of three dimensions: PA intensity (“What is the intensity of PA that you usually participate in?”), PA duration (“How long do you spend in each PA session?”), and PA frequency (“How often do you do PA every month/week?”). Each item is rated on a 5-point scale, where higher scores reflect a higher level of physical exercise. A composite total physical exercise score was calculated using the formula: Intensity score × (Duration score - 1) × Frequency score. The resulting score ranges from 0 to 100 points. Based on this score, Based on this composite score, participants were categorized into three PA levels: low ( ≤ 19 points), moderate (20-42 points), and high (≥43 points). The scale had a test-retest reliability of 0.82 and a Cronbach's α coefficient of 0.74, indicating acceptable reliability. To examine the structural validity of the scale in the current sample, a confirmatory factor analysis (CFA) was conducted. The model demonstrated an acceptable fit: the normed chi-square statistic (χ^2^/df) was 3.086, the comparative fit index (CFI) reached 0.976, and the Tucker-Lewis index (TLI) stood at 0.963. Regarding absolute fit, the goodness of fit index (GFI) was 0.991, the root mean square error of approximation (RMSEA) was 0.071. According to the criteria established by Schumacker and Lomax (2008), these indices confirm that the scale is well-suited for the study population.

#### 2.2.2 Peer Support Scale (PSS)

Peer support was assessed using Chen Niya's adapted version of the Peer Support Scale for College Students (Chen, 2007), which was based on Xiao Shuiyuan's original instrument. Items are rated on a 4-point scale (1-4) and summed, with higher total scores indicating greater perceived support. The scale demonstrated acceptable reliability and validity, with a Cronbach's α coefficient of 0.72. To examine the structural validity of the scale in the current sample, a confirmatory factor analysis (CFA) was conducted. The model demonstrated an acceptable fit: the normed chi-square statistic (χ^2^/df) was 3.042, the comparative fit index (CFI) reached 0.976, and the Tucker-Lewis index (TLI) stood at 0.960. Regarding absolute fit, the goodness of fit index (GFI) was 0.968, the root mean square error of approximation (RMSEA) was 0.070. According to the criteria established by Schumacker and Lomax (2008), these indices confirm that the scale is well-suited for the study population.

#### 2.2.3 Academic Self-Efficacy Scale (ASES)

Academic self-efficacy was assessed using the Academic Self-Efficacy Scale developed by Liang (2000). It consisted of 22 items. Responses are recorded on a 5-point Likert scale (1 = “completely disagree” to 5 = “completely agree”), with higher scores indicating a higher level of academic self-efficacy. The scale exhibited excellent internal consistency with a Cronbach's α coefficient of 0.96. A confirmatory factor analysis (CFA) was conducted to examine the structural validity of the scale. To improve model fit, residual covariances were introduced between four pairs of items (e.g., e1-e2, e16-e22, e18-e19, e20-e22) based on their shared content or methodological characteristics. The revised model yielded the following fit indices: χ^2^/df = 4.137, CFI = 0.952, TLI = 0.946, GFI = 0.840, and RMSEA = 0.087. Although the GFI and RMSEA were slightly below the conventional threshold of 0.90 and 0.08, respectively, the CFI and TLI exceeded the recommended 0.95 and 0.90 benchmarks. Overall, these results indicate that the scale's factor structure is largely acceptable for the current sample.

#### 2.2.4 Procrastination Assessment Scale-Students (PASS)

Academic procrastination was measured using the Procrastination Assessment Scale–Students (PASS), translated and adapted by Guan (2006). The scale assesses six typical academic tasks: writing term papers, studying for exams, completing weekly assignments, managing academic administrative duties, attending meetings, and performing general academic activities. For each task, three items evaluate procrastination behavior, its consequences, and the intention to change. All items are scored on a 5-point Likert scale (1-5). In this study, the first two items from each task were summed to create a total procrastination score, with higher scores reflecting more severe academic procrastination. The scale exhibited excellent internal consistency with a Cronbach's α coefficient of 0.93. A confirmatory factor analysis (CFA) was conducted to assess the structural validity of the Academic Procrastination Scale. The one-factor model showed inadequate fit. In line with the scale's theoretical dimensions, a correlated three-factor model (procrastination behavior, impact, and intention to change) was tested and significantly improved the fit. To account for residual covariances suggested by modification indices and substantive overlap in item content, eight pairs of error covariances were freed. The final model yielded an acceptable fit: χ^2^/df = 4.165, CFI = 0.932, TLI = 0.916, RMSEA = 0.087. These results support the proposed three-factor structure in the current sample.

### 2.3 Statistical analysis

This study adopted a dual-track analytical strategy combining the SPSS PROCESS macro and AMOS. In testing chained mediation mechanisms, prior research has predominantly employed the SPSS PROCESS macro, which has become a relatively well-established analytical approach in this field. In recent years, some studies have presented SPSS PROCESS results together with AMOS path diagrams to enhance the visual clarity of the findings. However, if the path coefficients are not independently estimated within the AMOS framework but are instead directly adopted from PROCESS results, these parameters do not originate from the structural equation modeling framework itself; therefore, such results should be interpreted with methodological caution. At the same time, the SPSS PROCESS macro typically aggregates latent variables into single observed scores and does not account for measurement error, which may affect the accuracy of parameter estimation (Sarstedt et al., 2020). By contrast, AMOS allows the simultaneous modeling of latent variables and their multiple indicators, thereby controlling for measurement error and providing a more precise estimation of the structural relationships among latent constructs (Hayes, 2009). In addition, AMOS uses maximum likelihood estimation and shows greater applicability in parameter estimation, standard error calculation, and overall model fit evaluation. Based on these considerations, the present study reported and compared the results from both approaches in order to ensure comparability with prior studies while further improving the accuracy and rigor of the findings. Given that AMOS allows latent variable modeling and accounts for measurement error, the AMOS results were treated as the primary basis for interpretation, whereas the PROCESS results were used as supplementary evidence.

## 3 Results

### 3.1 Common method bias test

A Harman's single-factor test was conducted to examine potential common method bias due to the self-report design (Podsakoff and Organ, 1986; Podsakoff et al., 2003). The results showed that the first unrotated factor explained 36.71% of the total variance, below the critical threshold of 40%, indicating that common method variance did not pose a substantial threat in this study. While this test provides a preliminary check, we recommend that future research adopt more robust designs, such as collecting multi-source data, implementing longitudinal assessments, or applying latent method factor modeling, to further control for such bias.

### 3.2 Demographic characteristics

As detailed in Table 1, the sample comprised 332 student-athletes. The majority were male (78.01%) and pursued sports-related majors (77.11%). Most were undergraduates (90.96%), with over half (60.54%) competing at the varsity level. Participants were nearly equally divided between individual (51.81%) and team sports (48.19%). The sample was primarily drawn from Eastern China (35.24%); most had no formal athlete rating (73.49%) and attended non-Double First-Class institutions (82.53%).

**Table 1:** Demographic and athletic characteristics of the sample (*N* = 332).

Characteristic	Category	*n*	*%*	Cumulative %
Gender	Male	259	78.01	78.01
	Female	73	21.99	100.00
Enrollment status	Associate degree student	2	0.60	0.60
	Undergraduate	302	90.96	91.57
	Master's student	25	7.53	99.10
	Doctoral student	3	0.90	100.00
Major	Sports major	256	77.11	77.11
	Non-Sports major	76	22.89	100.00
Varsity team member	Yes	201	60.54	60.54
	No	131	39.46	100.00
Sport type	Individual sport	172	51.81	51.81
	Team sport	160	48.19	100.00
University region	Eastern China	117	35.24	35.24
	Central China	58	17.47	52.71
	Western China	105	31.63	84.34
	Northeastern China	51	15.36	99.70
	Hong Kong, Macao, and Taiwan	1	0.30	100.00
Athlete grade level	No official grade	244	73.49	73.49
	Second-Grade athlete	49	14.76	88.25
	First-Grade athlete	36	10.84	99.10
	National master athlete	2	0.60	99.70
	International master athlete	1	0.30	100.00
Double first-class university	Yes	58	17.47	17.47
	No	274	82.53	100.00

### 3.3 Descriptive statistics

Table 2 presents the descriptive statistics for physical exercise (PE), peer support (PS), academic self-efficacy (ASE), and academic procrastination (AP) among student-athletes. The PE scale has a total score range of 0-100, with a mean score of 50.61 ± 25.41 for the participants. The PS scale is scored out of 40, and the average score was 24.16 ± 4.72, indicating a moderately high level of peer support. The ASE scale has a total score of 110, and the participants achieved a relatively high mean score of 87.63 ± 18.35, falling within the upper-medium range. In contrast, the AP scale (total score: 60) yielded a mean score of 27.06 ± 8.12, reflecting a moderate degree of academic procrastination. Overall, student-athletes reported strong peer support and high academic self-efficacy, yet they simultaneously exhibited a moderate level of academic procrastination. This pattern suggests a potential “confidence-action gap” in their academic lives. While high academic self-efficacy indicates their confidence in handling academic tasks, the persistence of procrastination may stem from challenges in time and energy management amid the dual demands of sports training and academic studies. The observed level of peer support likely serves as a protective resource, helping to buffer academic stress and facilitate their adaptation to these competing roles.

**Table 2:** Descriptive statistical analysis.

Variable	*N*	Mean	SD
PE	332	50.61	25.41
PS	332	24.16	4.718
ASE	332	87.63	18.35
AP	332	27.06	8.117

### 3.4 Correlation analysis of physical exercise, academic procrastination, peer support and self-efficacy in college student-athletes

**Figure 3:**
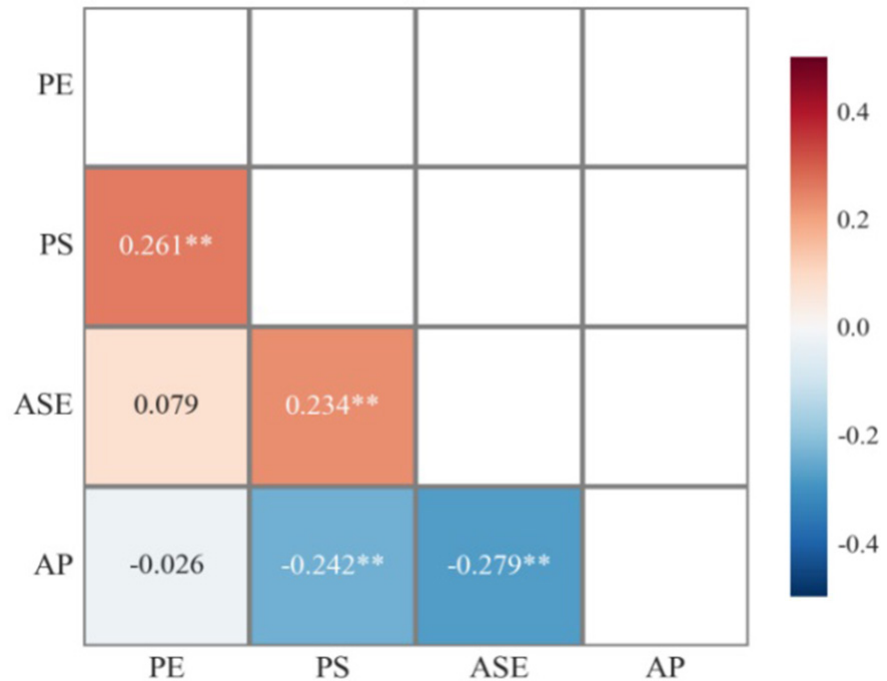
Structural equation modeling results.

A Pearson correlation analysis was conducted on the four variables—physical exercise (PE), peer support (PS), academic self-efficacy (ASE), and academic procrastination (AP) (see Figure 3) physical exercise (PE), peer support (PS), academic self-efficacy (ASE), and academic procrastination (AP). The analysis revealed that PS was significantly positively correlated with ASE (*r* = 0.234, *p* < 0.01) and significantly negatively correlated with AP (*r* = −0.242, *p* < 0.01). ASE was also significantly negatively correlated with AP (*r* = −0.279, *p* < 0.01). PE showed a significant positive correlation with PS (r = 0.261, *p* < 0.01). However, its correlations with ASE (*r* = 0.079, *p* > 0.05) and AP (*r* = −0.026, *p* > 0.05) were not statistically significant. These findings suggest that physical exercise was not directly associated with academic procrastination or academic self-efficacy at the zero-order level, and that its potential influence may instead operate through more complex indirect pathways. These results indicate that the core academic psychological variables (PS, ASE, and AP) are significantly interrelated in a theoretically expected pattern, while PE is specifically linked to PS. This correlation matrix provides a necessary foundation for further investigation into potential mediating mechanisms among these variables.

### 3.5 Chain mediation effect test

This study first conducted structural equation modeling using Amos 24.0 to examine the structural relationships among the variables. The model fit indices were as follows: χ^2^/df = 3.158, NFI = 0.793, IFI = 0.849, TLI = 0.839, CFI = 0.848, PNFI = 0.751, PCFI = 0.803, RMSEA = 0.081. All indices fell within acceptable ranges, indicating a good fit between the model and the data, thus providing preliminary support for the hypothesized path relationships among the variables.

To further examine the relationships among demographic variables, physical exercise, peer support, academic self-efficacy, and academic procrastination, hierarchical regression analyses were conducted. The results are presented in Table 3.

**Table 3:** Analysis of regression relationship of variables.

Variables	Peer support			Academic self-efficacy			Academic procrastination		
	*B*	*SE*	*t*	*B*	*SE*	*t*	*B*	*SE*	*t*
Gender	0.497	0.632	0.787	−0.761	2.512	−0.303	0.434	1.078	0.403
Enrollment status	0.520	0.779	0.667	−3.506	3.096	−1.133	2.317	1.331	1.741
Major	1.102	0.725	1.520	−3.607	2.892	−1.247	−0.674	1.244	−0.542
Varsity team member	−0.947	0.717	−1.321	−0.747	2.857	−0.262	−1.139	1.226	−0.929
Sport type	−0.949	0.531	−1.786	0.738	2.122	0.348	−0.708	0.911	−0.777
University region	−0.132	0.249	−0.528	−0.035	0.992	−0.036	−0.256	0.425	−0.602
Athlete grade level	0.019	0.437	0.043	2.302	1.738	1.324	−1.058	0.748	−1.416
Double first-class university	−0.209	0.796	−0.263	−0.299	3.162	−0.094	−1.049	1.356	−0.774
physical exercise	0.040	0.011	3.509	−0.013	0.046	−0.291	0.012	0.020	0.585
peer support	-			0.938	0.221	4.234	−0.378	0.098	−3.867
academic self-efficacy	-			-			−0.099	0.024	−4.143
F-value	4.439^**^			2.581^*^			4.432^**^		
R^2^	0.110			0.074			0.132		
Adjusted R^2^	0.086			0.046			0.102		

*B* = unstandardized regression coefficient.

^*^p<*0 .05;*

^**^p<*0 .01*.

For peer support, physical exercise demonstrated a significant and positive predictive effect (B = 0.040, *t* = 3.509, *p* < 0.01). This indicates that higher levels of physical exercise among student-athletes are associated with greater perceived peer support. None of the included demographic or contextual covariates significantly predicted peer support at the *p* < 0.05 level in this model. The overall model explained approximately 8.6% of the variance in peer support (adjusted *R*^2^ = 0.086).

For academic self-efficacy, physical exercise did not exhibit a significant direct effect (*B* = −0.013, *t* = −0.291, *p* > 0.05). However, peer support emerged as a strong and significant positive predictor (*B* = 0.938, *t* = 4.234, *p* < 0.01). This suggests that for student-athletes, peer support, rather than physical exercise, is a crucial factor associated with higher academic self-efficacy. None of the covariates reached statistical significance. This model accounted for about 7.4% of the variance in academic self-efficacy (*R*^2^ = 0.074).

Finally, for academic procrastination, both mediating variables showed significant predictive effects. Peer support had a significant negative relationship with procrastination (*B* = −0.378, *t* = −3.867, *p* < 0.01), as did academic self-efficacy (*B* = −0.099, *t* = −4.143, *p* < 0.01). This indicates that student-athletes who report stronger peer support and higher academic self-efficacy tend to engage in less academic procrastination. In contrast, the direct path from physical exercise to academic procrastination was non-significant (*B* = 0.012, *t* = 0.585, *p* > 0.05), suggesting that its influence is fully mediated through the proposed psychological and social variables. The covariates did not significantly predict procrastination in the final model. This comprehensive model explained 13.2% of the variance in academic procrastination (*R*^2^ = 0.132).

In summary, the results support a mechanism whereby physical exercise is positively linked to peer support, which in turn is associated with both higher academic self-efficacy and lower academic procrastination. The lack of significant direct effects from physical exercise to both academic self-efficacy and procrastination highlights the essential intervening roles of peer support and academic self-efficacy. These findings underscore the importance of fostering social support networks and enhancing self-efficacy beliefs within athletic programs to mitigate academic procrastination among student-athletes.

The bootstrap method with 5,000 resamples was further employed to test the mediating effects of peer support and academic self-efficacy in the relationship between physical exercise and academic procrastination, using 95% bias-corrected confidence intervals (CIs) (see Table 4). The results indicated that physical exercise had no significant direct effect on academic procrastination [direct effect = 0.012, 95% CI (−0.027, 0.050)]. However, the total indirect effect (the sum of all specified mediating pathways) was statistically significant [effect = −0.055, 95% CI (−0.115, −0.007)]. Decomposition of this total indirect effect revealed two significant mediating pathways: The simple mediation path through peer support alone was significant [physical exercise → peer support → academic procrastination: effect = −0.047, 95% CI (−0.099, −0.016)]. The sequential mediation path through both peer support and academic self-efficacy was also significant [physical exercise → peer support → academic self-efficacy → academic procrastination: effect = −0.012, 95% CI (−0.031, −0.003)]. In contrast, the simple mediation path through academic self-efficacy alone was not significant [effect = 0.004, 95% CI (−0.026, 0.034)]. Consequently, the total effect of physical exercise on academic procrastination was not statistically significant [total effect = −0.006, 95% CI (−0.046, 0.035)]. In summary, the results support a significant mediating mechanism wherein physical exercise is linked to reduced academic procrastination indirectly, primarily through enhancing peer support. This beneficial effect operates both through a direct path from peer support to lower procrastination, and through a sequential chain where peer support boosts academic self-efficacy, which in turn further reduces procrastination. The absence of a significant direct or total effect underscores the complete and critical mediating roles of these psychosocial factors.

**Table 4:** Direct effect, indirect effect and total effect among the variables (using SPSS).

Effect types	Item	Effect	SE	Boot 95%CI		Z
				LLCI	ULCI	
Direct effect	Physical exercise → Academic procrastination	0.012	0.020	−0.027	0.050	0.586
Indirect effect	Physical exercise → Peer support → Academic procrastination	−0.047	0.020	−0.099	−0.016	−2.337
	physical exercise → Academic self-efficacy → Academic procrastination	0.004	0.015	−0.026	0.034	0.282
	Physical exercise → Peer support → Academic self-efficacy → Academic procrastination	−0.012	0.007	−0.031	−0.003	−1.785
Total indirect effect	Physical exercise → Peer support → Academic procrastination+Physical exercise → Academic self-efficacy → Academic procrastination+Physical exercise → Peer support → Academic self-efficacy → Academic procrastination	−0.055	0.027	−0.115	−0.007	−2.011
Total effect	Total indirect effect + Direct effect	0.006	0.020	−0.046	0.035	0.586

**Figure 4:**
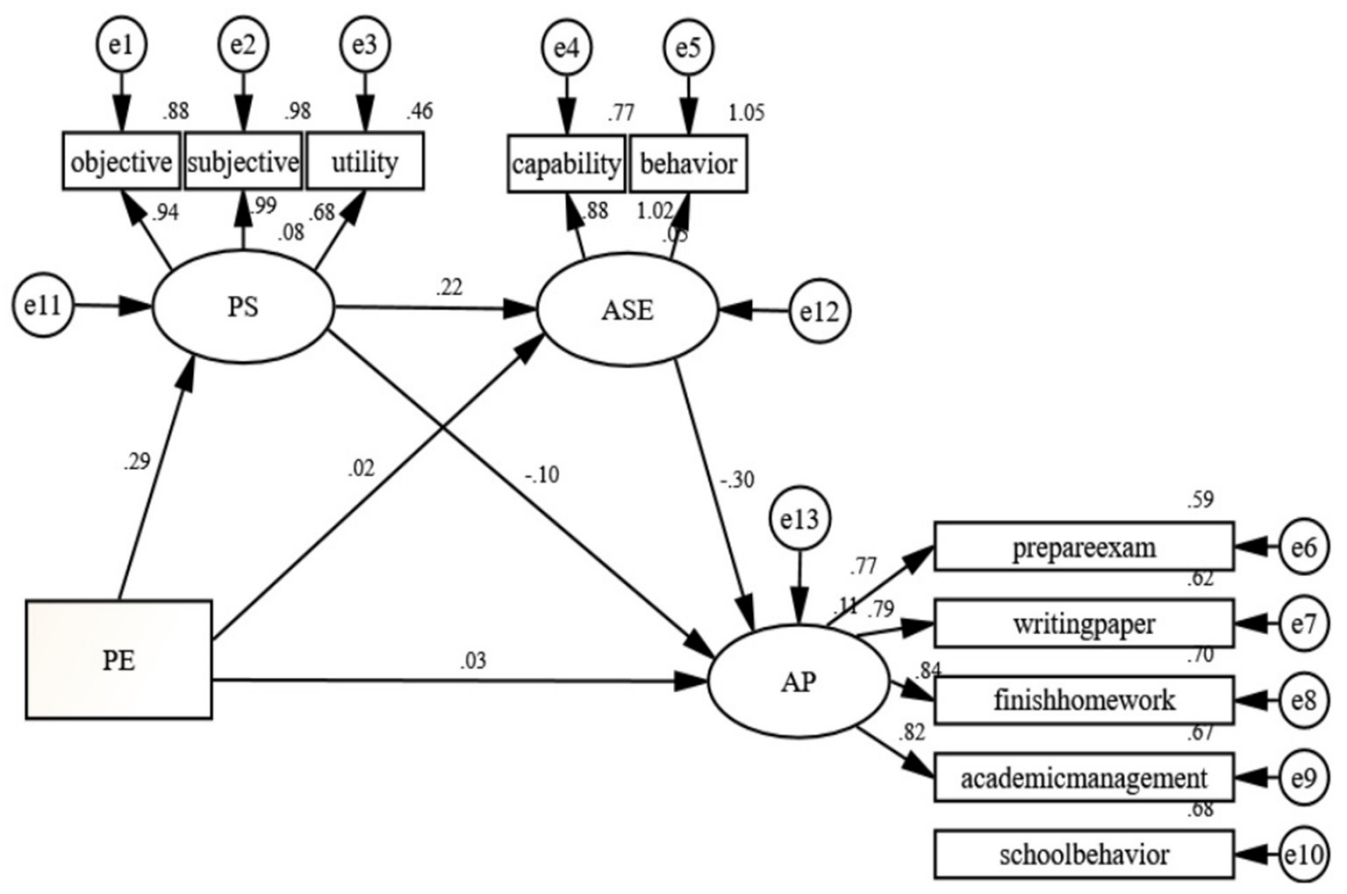
Bootstrapped indirect effects.

When constructing the model, the independent variable physical activity was treated as an observed variable, derived by multiplying three items. Peer support, as a mediator, was divided into three dimensions: objective support, subjective support, and support utilization. Another mediator, academic self-efficacy, was conceptualized across two dimensions: capability and behavior. The dependent variable, academic procrastination, was categorized into six dimensions. The specific structure is shown in Figure 4.

The results revealed that the direct effect of physical exercise on academic procrastination was not significant [direct effect = 0.033, 95% CI (−0.079, 0.140)]. However, the total indirect effect, the sum of all specified mediating pathways, was statistically significant [effect = −0.053, 95% CI (−0.110, −0.008)]. Further decomposition of the indirect effects indicated one significant mediating pathway: the sequential mediation through both peer support and academic self-efficacy was significant [physical exercise → peer support → academic self-efficacy → academic procrastination: effect = −0.019, 95% CI (−0.041, −0.006)]. In contrast, neither the simple mediation through peer support alone [effect = −0.029, 95% CI (−0.073, 0.004)] nor the simple mediation through academic self-efficacy alone [effect = −0.005, 95% CI (−0.049, 0.025)] reached statistical significance. Consequently, the total effect of physical exercise on academic procrastination was not significant [total effect = −0.019, 95% CI (−0.135, 0.094)]. In summary, these findings support a significant sequential mediation mechanism in which physical exercise is associated with reduced academic procrastination indirectly by enhancing peer support, which in turn increases academic self-efficacy, thereby further decreasing procrastination (see Table 5).

**Table 5:** Direct effect, indirect effect and total effect among the variables (using AMOS).

Effect types	Item	Effect	SE	Boot 95%CI		Z
				LLCI	ULCI	
Direct effect	Physical exercise → Academic procrastination	0.033	0.056	−0.079	0.14	0.589
Indirect effect	Physical exercise → Peer support → Academic procrastination	−0.029	0.019	−0.073	0.004	−1.526
	physical exercise → Academic self-efficacy → Academic procrastination	−0.005	0.018	−0.049	0.025	−0.278
	Physical exercise → Peer support → Academic self-efficacy → Academic procrastination	−0.019	0.009	−0.041	−0.006	−2.111
Total indirect effect	Physical exercise → Peer support → Academic procrastination+Physical exercise → Academic self-efficacy → Academic procrastination+Physical exercise → Peer support → Academic self-efficacy → Academic procrastination	−0.053	0.026	−0.11	−0.008	−2.038
Total effect	Total indirect effect + Direct effect	−0.019	0.059	−0.135	0.094	−0.322

It is evident that the two analytical approaches yielded different patterns of significant pathways. Hypothesis 4 was supported under both statistical methods, indicating that physical exercise indirectly reduced academic procrastination by enhancing peer support, which in turn strengthened academic self-efficacy. In contrast, the simple mediating effect of peer support alone was supported only by the SPSS PROCESS macro. In other words, Hypothesis 2 was supported under the SPSS PROCESS analysis but not under the AMOS analysis. These results collectively demonstrate that physical exercise does not directly reduce academic procrastination among student-athletes, nor does it reduce procrastination solely by enhancing peer support or academic self-efficacy. Instead, its beneficial effect is fully mediated by a sequential mechanism: physical exercise first strengthens peer support, which in turn promotes academic self-efficacy, ultimately leading to a reduction in academic procrastination.

## 4 Discussion

The direct path (H1) was not supported. The study found that the direct negative predictive effect of physical exercise on academic procrastination was not significant. This result suggests that mere physical engagement may not directly translate into self-regulatory capacity in the academic domain. Student-athletes often experience role conflict between training and academic responsibilities, whereby physical exercise can be constructed as a “professional activity” distinct from their academic identity. Consequently, the physiological arousal effects of exercise cannot cross role boundaries to influence cognitive task strategies (Maciá-Andreu et al., 2023). In the sports domain, these individuals exhibit stronger competence needs, greater willingness to exert effort, and a stronger tendency to attribute success to personal effort (Dishman et al., 2006). This finding is not consistent with previous research among Chinese college students, which reported that physical activity had a significant negative effect on academic procrastination (Ren et al., 2021). A possible explanation is that, for student-athletes, physical exercise is a routine and role-related activity rather than an additional health-promoting behavior. Therefore, its direct influence on academic procrastination may be weaker than that observed in general college student populations.

It should be noted that the absence of significant zero-order correlations between physical exercise and academic procrastination, as well as between physical exercise and academic self-efficacy, does not necessarily preclude the existence of significant indirect effects. In the present study, physical exercise was theorized to influence academic procrastination primarily through a sequential pathway involving peer support and academic self-efficacy, rather than through a direct mechanism. From the perspective of social cognitive theory, behavioral factors may first shape environmental resources, such as peer support, which then influence personal cognitive factors, such as academic self-efficacy, and ultimately affect academic behavior. Therefore, the serial mediation model was tested on theoretical rather than purely correlational grounds.

The hypothesized mediation pathway via peer support (H2) was not supported. The results of this study reveal that, among student-athletes, peer support did not significantly mediate the relationship between physical exercise and academic procrastination. While exercise likely enhances social interaction, such peer engagement did not translate into reduced academic delay. This may be explained by the domain-specific nature of their support systems: peer networks are oriented toward athletic performance and are not readily activated in academic contexts (Burns et al., 2013). Moreover, a strong athletic identity often coincides with a weaker student identity. This role conflict means that team-based support seldom transfers to the student role, thereby failing to promote academic self-regulation (Lally and Kerr, 2005). This result also differs from previous studies showing that peer-related social resources can promote positive behavioral intentions through social support and self-efficacy pathways (Zhou et al., 2025). However, that study focused on general college students' intention to engage in physical activity, whereas the present study examined academic procrastination among student-athletes, suggesting that the function of peer support may vary across behavioral domains and populations.

Mediation pathway via academic self-efficacy (H3) was not supported. No significant path was found from physical exercise to academic procrastination via academic self-efficacy. This indicates a “compartmentalization of self-efficacy” (Bong, 2001) in student-athletes. Our results suggest that the high competence fostered in sports does not readily transfer to academic settings, likely because the cognitive schemas and demands differ between these two domains. This result is also inconsistent with previous findings showing that self-efficacy significantly mediated the relationship between physical activity and academic procrastination among Chinese college students (Ren et al., 2021). One possible explanation is that the present study focused on student-athletes, whose efficacy beliefs may be more domain-specific. In this group, the influence of physical exercise on academic procrastination may require activation through social resources such as peer support before being translated into academic self-efficacy.

The hypothesized chain mediation pathway (H4) was supported. Key findings indicate that physical exercise first enhances peer support, which then boosts academic self-efficacy, and ultimately reduces academic procrastination. This pathway reveals that: (1) Peer support fosters a psychologically safe environment for reshaping academic self-efficacy, wherein sharing academic experiences within the team helps individuals reframe their perceptions of academic capabilities. (2) The culture of mutual accountability developed during training extends into the academic domain, facilitating a shift from external supervision to self-monitoring. (3) Moreover, the sense of belonging within the team promotes identity integration, which alleviates role conflict between “athlete” and “student,” thereby laying the groundwork for developing a positive academic self-concept.

The SPSS PROCESS macro and AMOS yielded different results in the mediation analysis. The AMOS structural equation modeling results may provide a more rigorous basis for interpretation, as this approach allows latent variable modeling and accounts for measurement error. Therefore, it is recommended that future studies of this kind make greater use of AMOS and report the path coefficients estimated from the AMOS analysis.

## 5 Research implication

### 5.1 Implementing a peer support mechanism

To address the academic heterogeneity among student-athletes, a tiered peer support system can be integrated within team structures. This involves preferentially pairing teammates from the same or related majors for discipline-specific collaboration, while cross-disciplinary partnerships should focus on general academic supervision. The latter includes shared time planning, progress monitoring, and exchange of learning strategies, explicitly excluding subject-specific instruction. A brief structured academic check-in during weekly team meetings allows members to report key upcoming tasks and request specific support, such as joint study sessions or resource referrals, thereby mobilizing team accountability. Furthermore, implementing a shared academic calendar in team spaces that displays all members' major deadlines leverages group awareness to create a culture of collective oversight. This mechanism channels team cohesion into process-focused support and habit formation, effectively navigating around barriers posed by disciplinary diversity.

### 5.2 Establishing an academic captain

Enhancing the precision and sustainability of academic support requires appointing an “Academic Captain” within each team. This role should be assigned to an academically strong and skilled member who receives targeted training. The training equips them to identify teammates' academic needs, facilitate constructive academic discussions within the team. Organizing regular forums for academic captains across different teams fosters the exchange of effective practices and common solutions, building a cross-team academic support network. This initiative formalizes informal peer help into a structured and enduring system. It enables student-athletes to navigate academic challenges more efficiently and cultivates a collective culture that visibly values and promotes academic success.

### 5.3 Research limitations and future directions

While this study offers valuable insights, several limitations should be noted.

First, the cross-sectional design restricts the ability to establish causal inferences among physical exercise, peer support, academic self-efficacy, and academic procrastination. Although significant correlations were observed between some variables, longitudinal or experimental research is needed to further verify the direction of causality.

Second, the physical activity scale employed in this study was primarily designed for the general university student population. Although student-athletes share a student identity, their physical activity levels are typically substantially higher than those of non-athlete students, which leads to uneven data distribution and consequently affects the stability of the results derived from the current model. Future research should consider developing a specialized and more discriminative physical activity scale tailored to student-athletes to more accurately capture the relationships among the variables.

Third, this study did not incorporate an intervention component and was limited to analyzing associations between variables, thus failing to assess actual intervention effects. Subsequent studies could introduce intervention experiments to evaluate the specific effects of physical exercise on mitigating procrastination behaviors, thereby providing more practical and actionable guidance.

Fourth, the explanatory power of the regression models was modest, with *R*^2^ values ranging from 0.074 to 0.132 and adjusted *R*^2^ values ranging from 0.046 to 0.102. This indicates that the predictors included in the present study explained only a limited proportion of variance in the outcome variables. Accordingly, the findings should be interpreted cautiously, and stronger causal or practical claims should be made with restraint.

## 6 Conclusion

This study integrates psychological mechanisms, namely peer support and academic self-efficacy, into the investigation of the relationship between physical exercise and academic procrastination among student-athletes. The findings confirm a chain mediation pathway in which physical exercise enhances peer support, which in turn strengthens academic self-efficacy and ultimately reduces academic procrastination. In addition, the comparison between SPSS PROCESS macro and AMOS indicated that different analytical approaches may yield different mediation results. This finding highlights the importance of carefully selecting and reporting statistical methods in future research on chained mediation models.

## Data Availability

The original contributions presented in the study are included in the article/supplementary material, further inquiries can be directed to the corresponding author.
